# Assessing the role of statin therapy in bladder cancer: evidence from a Mendelian Randomization study

**DOI:** 10.3389/fphar.2024.1427318

**Published:** 2024-07-19

**Authors:** Rongkang Li, Guixiao Huang, Yunfei Li, Mou Huang, Ying Huang, Yingrui Li, Guangzhi Li, Song Wu

**Affiliations:** ^1^ Institute of Urology, Lanzhou University Second Hospital, Lanzhou University, Lanzhou, China; ^2^ Institute of Urology, The Affiliated Luohu Hospital of Shenzhen University, Shenzhen University, Shenzhen, China; ^3^ Institute of Urology, South China Hospital, Health Science Center, Shenzhen University, Shenzhen, China

**Keywords:** Mendelian Randomization, statin, bladder cancer, causal analysis, GWAS

## Abstract

**Background:**

Statins, which are medications that lower lipid levels, are extensively used to decrease cardiovascular disease risk. Recently, the use of statins in cancer prevention has attracted considerable interest. However, it is still unclear whether the use of statins has a causal effect on bladder cancer.

**Methods:**

The two-sample Mendelian Randomization (MR) was performed to infer the causal relationship between statin therapy (atorvastatin, simvastatin, and rosuvastatin) and bladder cancer. Single-nucleotide polymorphisms (SNP)-based genome-wide association studies (GWAS) of statins (atorvastatin, simvastatin, and rosuvastatin) were gathered from the UK Biobank, involving 462,933 participants. We acquired summary-level genetic data on bladder cancer from a European cohort of 175,121 individuals. The inverse variance weighted (IVW) method was the main analytical technique used, supplemented by MR-Egger, weighted median, weighted mode, and simple mode to estimate causal effects. Additionally, sensitivity analyses were conducted to verify the robustness and reliability of our findings.

**Results:**

Based on the IVW analysis, we identified a significant causal association between rosuvastatin use and a decreased risk of bladder cancer, with genetic analysis inferring the substantial reduction in odds (OR = 3.52E-19, 95% CI: 5.48E-32–2.26E-06, *p* = 0.005). In contrast, the IVW results did not reveal a statistically significant relationship between the genetically estimated use of atorvastatin (OR = 7.42E-03, 95% CI: 6.80E-06–8.084, *p* = 0.169) or simvastatin (OR = 0.135, 95% CI: 0.008–2.330, *p* = 0.168) and bladder cancer risk.

**Conclusion:**

We investigated the causal link between statin therapy (atorvastatin, simvastatin, and rosuvastatin) and bladder cancer using a two-sample Mendelian Randomization analysis among the European population. Our findings indicated that genetically predicted use of rosuvastatin was associated with a decreased risk of bladder cancer, whereas no significant genetically predicted causal effects were observed for atorvastatin and simvastatin use.

## 1 Introduction

Bladder cancer (BCa), frequently diagnosed as a malignant urological tumor, originates mainly from malignant transitional epithelial cells (Dyrskjot et al., 2023). In 2022, this cancer accounted for 84,825 new cases in the United States and 91,893 in China, while Europe reported 204,000 new cases in 2020 (Dyba et al., 2021; Xia et al., 2022). Known as the costliest malignancy to manage, bladder cancer poses a significant healthcare challenge due to its tendency for frequent relapses and the static nature of treatment advancements, necessitating expensive ongoing monitoring and multiple interventions (Leal et al., 2016; Richters et al., 2020). In the United States, the annual total cost of cancer was $183 billion in 2015, and it is projected to increase to $246 billion by 2030. Among these costs, the annual medical burden of bladder cancer was approximately $7.93 billion, with an expected increase to $11.6 billion by 2030 (Mariotto et al., 2020). Similarly, in the European Union member states, the total cost of cancer was €152.8 billion in 2012, with the medical burden of bladder cancer accounting for approximately €5.24 billion (Leal et al., 2016). Effective early prevention, screening, and accurate diagnosis are pivotal in lessening the burden of this disease on society (Lenis et al., 2020). In the realm of cancer prevention, particularly through chemoprevention, medications commonly used for metabolic and cardiovascular conditions have been noticed for their beneficial impacts on the anticancer process (Gronich and Rennert, 2013; Morales and Morris, 2015).

Statins, which are 3-Hydroxy-3-methylglutaryl-coenzyme A reductase (HMGCoAR) inhibitors, effectively reduce lipids and are the primary treatment for hypercholesterolemia by blocking liver-based endogenous cholesterol production (Igel et al., 2002; Ziaeian and Fonarow, 2017). These medications might also serve a chemopreventive function against cancer, as a decrease in cholesterol could restrict the cell proliferation necessary for cancer development and spread (Rosch and McCully, 2013). As the critical enzyme in the mevalonate pathway, HMGCoAR supports essential cellular growth and survival processes (Mullen et al., 2016). Furthermore, statins are known to obstruct Ras/Rho pathways, thereby curtailing various cancer-promoting signaling routes (Ahmadi et al., 2017; Patel and Kashfi, 2022). A nested case-control study within the UK Clinical Practice Research Datalink (CPRD) found that current statins use correlates with a 12% reduction in the risk of biliary tract cancers compared to non-use (Liu et al., 2019). However, few randomized controlled trials (RCTs) have been conducted to evaluate the effect of statins on bladder cancer (Symvoulidis et al., 2023).

Mendelian randomization (MR) is an analytical method increasingly employed to determine causal relationships between exposures and outcomes (Davies et al., 2018). This technique uses genetic variations as instrumental variables (IV) to firmly establish causality between exposures and outcomes (Davey Smith and Hemani, 2014; Burgess et al., 2015). By leveraging the random distribution of these genetic variations, MR effectively minimizes the impact of confounding factors and reverse causality. This approach emulates the randomization seen in RCTs, thereby circumventing the confounding effects and potential biases associated with traditional RCTs (Lawlor et al., 2008). Utilizing data from Genome-wide association studies (GWAS), MR has been extensively applied in various public health sectors (Sekula et al., 2016; Hemani et al., 2018). Accordingly, a two-sample MR method was used to investigate the causal link between statin usage and bladder cancer, thus providing a more robust basis for clinical decision-making through GWAS data insights.

## 2 Methods

### 2.1 Study design and ethics statement

**FIGURE 1:**
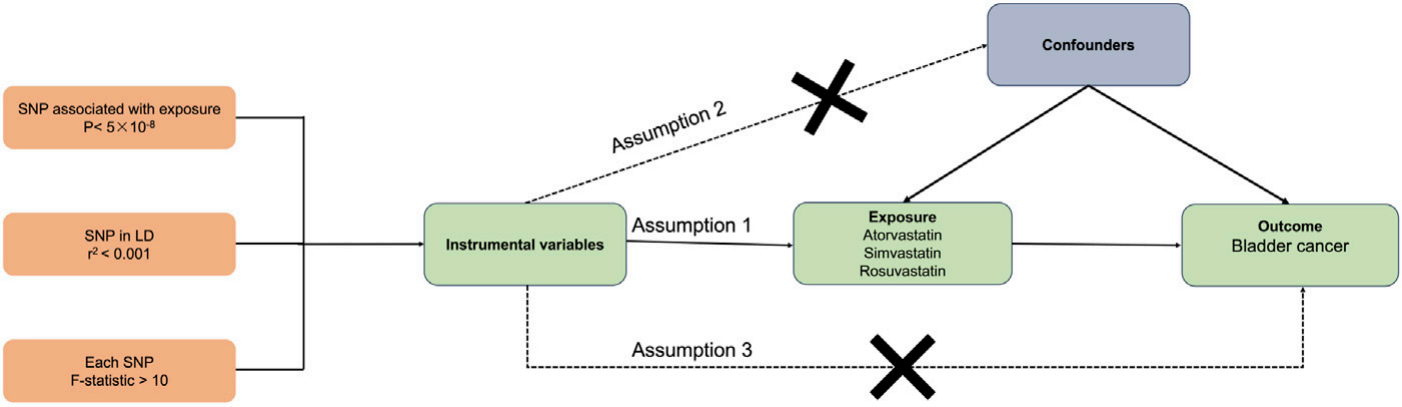
Overall design of this MR analyses.

In this study, MR analysis was utilized to explore the causal relationship between statin use and bladder cancer (Figure 1). MR analysis is based on public GWAS data, and we utilized publicly available GWAS datasets for atorvastatin, simvastatin, and rosuvastatin usage in this MR analysis. The use of atorvastatin, simvastatin, and rosuvastatin served as the exposure variables in our study. The study design and reporting conformed to using STROBE-MR (Skrivankova et al., 2021a; Skrivankova et al., 2021b). MR analysis employs instrumental variables (IVs) to evaluate causal links between exposures and outcomes. This method hinges on three critical assumptions (Lawlor et al., 2008). The first assumption requires that the genetic variants (single-nucleotide polymorphisms, SNPs) used as IVs must have a strong association with the exposure (statins). The second assumption states that these genetic variants should not be linked with any confounders affecting the relationship between the exposure and outcomes. Finally, the third assumption mandates that the genetic variants should influence the outcome solely through their effect on the exposure, excluding any other indirect pathways. Since our data were sourced from previously conducted research and publicly available databases, obtaining further ethical approval from an ethics committee was not necessary.

### 2.2 Data source

We obtained summary statistics for statin use and bladder cancer from the International Oncology Unit (IEU) Open GWAS project (https://gwas.mrcieu.ac.uk/datasets/). The data for atorvastatin, simvastatin, and rosuvastatin came from the MRC-IEU consortium (Bycroft et al., 2018; Hemani et al., 2018). Specifically, the atorvastatin dataset (ukb-b-10008) included 13,851 cases and 449,082 controls; the simvastatin dataset (ukb-b-11268) included 52,427 cases and 410,506 controls; and the rosuvastatin dataset (ukb-b-13664) included 2,870 cases and 460,063 controls. To avoid population overlap in the exposure and outcome assessments, we sourced GWAS summary-level data linked to BCa from a FinnGen biobank cohort of European descent (1,115 cases and 174,006 controls) via the IEU Open GWAS database (Kurki et al., 2023). We exclusively used datasets of European ancestry to mitigate bias due to population stratification. Detailed information about these four GWAS datasets is available in Table 1.

**TABLE 1:** Characteristics of statin (atorvastatin, simvastatin, and rosuvastatin) use and bladder cancer GWAS cohorts in this study.

Exposure/outcome	IEU GWAS id	Cases	Controls	Sample size	Number of SNPs	Population	Consortium	Year
Atorvastatin use	ukb-b-10008	13,851	449,082	462,933	9,851,867	European	MRC-IEU	2018
Simvastatin use	ukb-b-11268	52,427	410,506	462,933	9,851,867	European	MRC-IEU	2018
Rosuvastatin use	ukb-b-13664	2,870	460,063	462,933	9,851,867	European	MRC-IEU	2018
Bladder cancer	finn-b-C3_BLADDER_EXALLC	1,115	174,006	175,121	16,380,305	European	FinnGen study	2021

### 2.3 Selection of instrumental variables

Drawing from the GWAS summary data mentioned earlier, a rigorous procedure was followed to select suitable SNPs as IVs. Initially, SNPs had to demonstrate a strong association with the exposure, achieving a genome-wide significance with a *p*-value < 5 × 10^−8^. Secondly, to prevent results skewed by linkage disequilibrium (LD), a clumping process was implemented with an *R* ^2^ cutoff of 0.001 and a window size of 10,000 kb. Thirdly, the Phenoscanner database (http://www.phenoscanner.medschl.cam.ac.uk/) was employed to identify genetic variants linked to potential confounders. SNPs closely related to the potential confounders, including smoking, body mass index, waist-to-hip ratio, and type 2 diabetes mellitus were excluded (*p*-value < 5 × 10^−8^). The remaining SNPs were correspondingly chosen as IVs for exposes (Cheng et al., 2023). Fourthly, if these specific SNPs were absent in the outcome GWAS dataset, proxy SNPs (with a high LD, *R* ^2^ > 0.8, with the target SNPs) were sought as alternatives. Finally, to ensure the consistency of effect alleles between the exposure and outcome datasets, harmonization was carried out to exclude palindromic and ambiguous SNPs with non-matching alleles. Additionally, to robustly adhere to the first key assumption, the F-statistic for each SNP was calculated individually; SNPs with F statistics < 10 were deemed weak IVs and excluded from further analysis (Burgess et al., 2011; Li et al., 2023; 2024). Following these stringent filters, the selected SNPs were utilized as the definitive IVs for the ensuing two-sample MR study.

### 2.4 Statistical analysis

In our research, we utilized a range of methods to analyze the causal connections and effects between exposure and outcome, including inverse-variance weighted (IVW) (Burgess et al., 2016), MR-Egger (Bowden et al., 2015), weighted median (Bowden et al., 2016), weighted mode (Hartwig et al., 2017), and simple mode (Zhang et al., 2023). Each method is suited to different scenarios. The IVW method uses SNPs’ Wald estimators to determine the influence of exposure on outcome. We primarily rely on the IVW approach when there is no pleiotropy, or when pleiotropy is balanced, to derive reliable causal estimates. If significant heterogeneity among the IVs was detected (*p* < 0.05), a random effects model was applied; otherwise, a fixed effects model was employed (Burgess et al., 2016). The MR-Egger regression provides credible estimates under conditions of IV pleiotropy (Bowden et al., 2015). The Weighted Median method, not requiring the Instrument Strength Independent of Direct Effect (InSIDE) assumption, offers significant improvements over MR-Egger by achieving unbiased effect estimates and lower type I error through evaluation of the weighted median of instrumental variable ratio estimate (Bowden et al., 2016). The weighted mode method is effective for MR causal inference assuming most IVs are valid (Hartwig et al., 2017), while the simple mode method generally yields less robust results compared to IVW(Zhang et al., 2023). All these methods were executed and visually presented using R version 4.3.1 with the “MRPRESSO” and “TwoSampleMR” packages, considering a *p*-value under 0.05 as statistically significant.

### 2.5 Sensitivity analysis

We conducted MR-Egger regression to evaluate pleiotropy in the instrumental variables, considering pleiotropy confirmed when the *p*-value fell below 0.05. We also implemented the MR-PRESSO test to further assess pleiotropy and identify outliers. In instances where the MR-PRESSO test revealed significant horizontal pleiotropy, we removed the implicated outlier variants and repeated the MR analysis. To quantify heterogeneities uncovered by both the IVW and MR-Egger regression methods, we calculated Cochran’s Q statistic, with a *p*-value of less than 0.05 indicating significant heterogeneity. Additionally, we executed a “leave-one-out” sensitivity analysis to ascertain whether any single SNP could significantly skew the overall causal inference.

## 3 Results

### 3.1 Selection of instrumental variables

Adhering to strict criteria for instrumental SNP selection, we identified appropriate SNPs as IVs that met three key assumptions. We identified 22 SNPs highly correlated with atorvastatin use, 39 SNPs highly correlated with simvastatin use, and 6 SNPs highly correlated with rosuvastatin use. These SNPs served as IVs for exposure, with each SNP displaying an F-statistic greater than 10, indicating a minimal likelihood of weak IV bias. Detailed descriptions of the included SNPs are provided in the Supplementary Tables S1-S3.

### 3.2 Causal effects of atorvastatin use on bladder cancer

**FIGURE 2:**
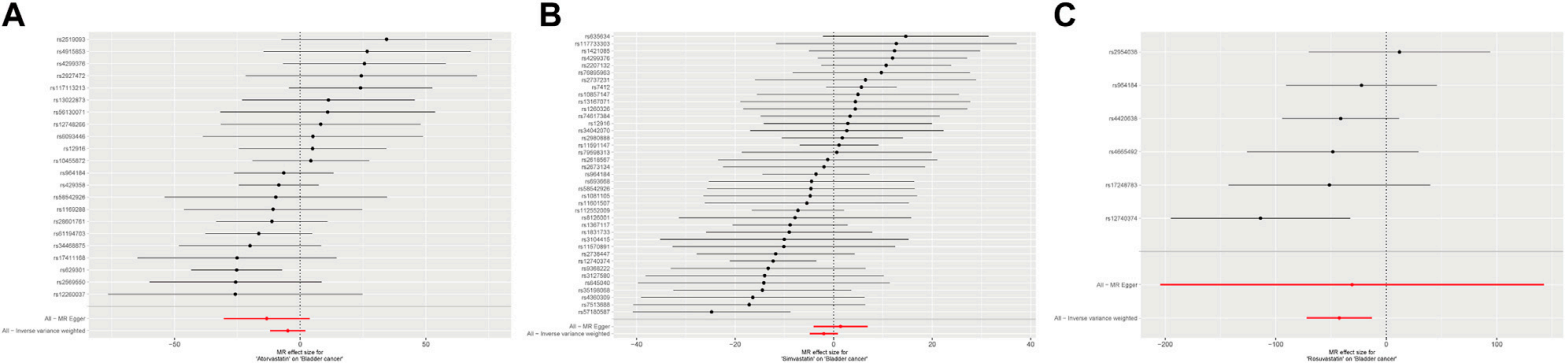
Forest plot of the causal effects of atorvastatin **(A)**, simvastatin **(B)**, and rosuvastatin **(C)** use associated SNPs on bladder cancer.

**FIGURE 3:**
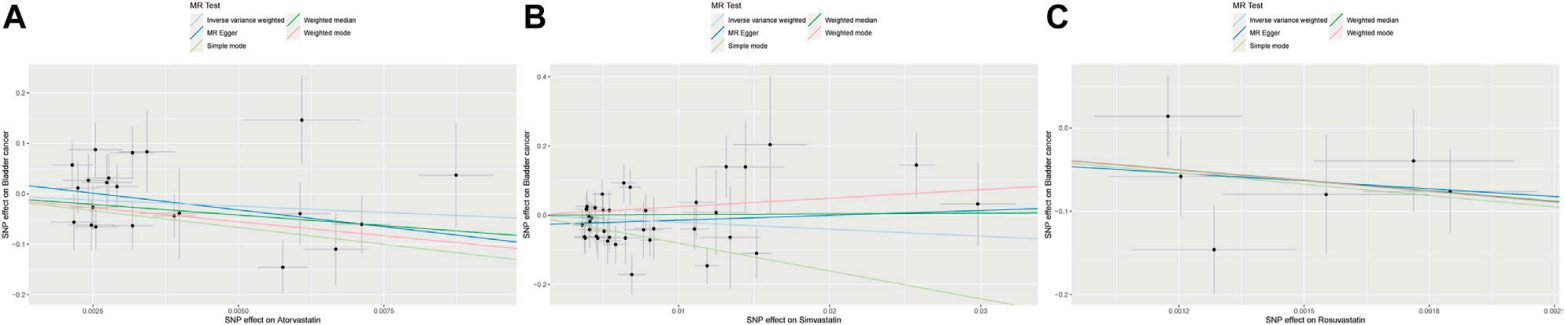
Scatter plot of the causal relationships between atorvastatin **(A)**, simvastatin **(B)**, and rosuvastatin **(C)** use on bladder cancer. The regression slopes of the lines represent the magnitude of the causal effect.

Table 2 displayed the outcomes of the MR analysis on the causal effects of atorvastatin use on bladder cancer. The inverse variance weighted (IVW) method revealed no causal connection between atorvastatin use and bladder cancer (OR = 7.42E-03, 95% CI: 6.80E-06–8.084, *p* = 0.169), a finding supported by additional methods including MR-Egger, Weighted Median, Weighted Mode, and Simple Mode. These results were graphically represented in the forest plot (Figure 2) and the scatter plot (Figure 3). The forest plot delineated the effect estimates and their confidence intervals for each SNP, while the scatter plot illustrated the relationship between atorvastatin use and bladder cancer using the instrumental variables. Thus, our analysis indicated that there was no significant causal effect of atorvastatin use on bladder cancer.

**TABLE 2:** MR analysis of the causality of statin (atorvastatin, simvastatin, and rosuvastatin) use on Bladder cancer.

Exposure	Outcome	MR method	Number of SNPs	β	SE	OR (95% CI)	*p*-value
Atorvastatin use	Bladder cancer	MR Egger	22	−13.291	8.704	1.69E-06 (6.59E-14–41.313)	0.142
		Weighted median	22	−8.413	4.535	2.22E-04 (3.06E-08–1.608)	0.064
		Inverse variance weighted	22	−4.904	3.568	7.42E-03 (6.80E-06–8.084)	0.169
		Simple mode	22	−13.347	9.318	1.60E-06 (1.87E-14–136.345)	0.167
		Weighted mode	22	−11.072	6.166	1.55E-05 (8.77E-11–2.754)	0.087
Simvastatin use	Bladder cancer	MR Egger	39	1.400	2.795	4.055 (0.017–970.731)	0.619
		Weighted median	39	0.209	2.134	1.232 (0.019–80.700)	0.922
		Inverse variance weighted	39	−2.002	1.453	0.135 (0.008–2.330)	0.168
		Simple mode	39	−8.037	5.378	3.23E-04 (8.54E-09–12.231)	0.143
		Weighted mode	39	2.464	4.407	11.756 (0.002–6.63E+04)	0.579
Rosuvastatin use	Bladder cancer	MR Egger	6	−30.950	88.525	3.61E-14 (1.60E-89–8.18E+61)	0.744
		Weighted median	6	−41.864	19.141	6.59E-19 (3.36E-35–0.013)	0.029
		Inverse variance weighted	6	−42.490	15.047	3.52E-19 (5.48E-32–2.26E-06)	0.005
		Simple mode	6	−45.048	27.191	2.73E-20 (1.95E-43–3,810.426)	0.158
		Weighted mode	6	−42.213	24.370	4.65E-19 (8.73E-40–257.729)	0.144

SNPs, single-nucleotide polymorphisms; SE, standard error; OR, odds ratio; CI, confidence interval

Additionally, the MR-Egger regression intercept analysis (*p* = 0.304) and the MR-PRESSO global test (*p* = 0.121) did not show significant pleiotropy (Table 3). The Cochran Q-test results from both the MR-Egger (*p* = 0.137) and IVW (*p* = 0.128) methods also demonstrated no heterogeneity in our findings. The funnel plots, presented in Supplementary Figure S1. Furthermore, the leave-one-out analysis verified that excluding any single SNP did not significantly influence the estimated causal relationship (Supplementary Figure S2).

**TABLE 3:** Sensitivity analyses of MR.

Exposure	Pleiotropy					Heterogeneity			
	MR-PRESSO global outlier test			MR-egger regression		MR-egger		Inverse variance weighted (IVW)	
	*p*-value	Outlier	*p*-value after outlier	Intercept	*p*-value	Q statistic	*p*-value	Q statistic	*p*-value
Atorvastatin use	0.121	—	—	0.035	0.304	26.927	0.137	28.428	0.128
Simvastatin use	0.039	No significant outliers	NA	−0.028	0.164	48.641	0.095	51.285	0.074
Rosuvastatin use	0.487	—	—	−0.017	0.901	5.026	0.285	5.048	0.410

### 3.3 Causal effects of simvastatin use on bladder cancer

The results from the MR analysis investigating the causal effects of simvastatin on bladder cancer were presented in Table 2. The IVW method indicated that simvastatin use had no causal impact on bladder cancer (OR = 0.135, 95% CI: 0.008–2.330, *p* = 0.168), a conclusion corroborated by the MR-Egger, Weighted Median, Weighted Mode, and Simple Mode analyses. Figures 2, 3 respectively illustrated these findings through a forest plot and a scatter plot, reinforcing the lack of a significant causal relationship between simvastatin use and bladder cancer.

Further evaluations using the MR-Egger regression intercept analysis for assessing pleiotropy among the instrumental variables showed no significant pleiotropic effects (*p* = 0.164) (Table 3). Although the *p*-value of MR-PRESSO global test was less than 0.05, MR-PRESSO global test showed that no significant outliers. The outlier-corrected causal estimate showed the NA. Heterogeneity assessments conducted through the MR-Egger (*p* = 0.095) and IVW (*p* = 0.074) methods also indicated no significant heterogeneity among the instrumental variables. The funnel plots depicted in Supplementary Figure S1. Additionally, the leave-one-out analysis verified the stability of the causal association estimate, demonstrating that it was unaffected by the exclusion of any individual SNP (Supplementary Figure S2).

### 3.4 Causal effects of rosuvastatin use on bladder cancer

The outcomes of the MR analysis of the causality of rosuvastatin use on bladder cancer were encapsulated in Table 2. IVW revealed a statistically significant negative causal impact of A rosuvastatin use on bladder cancer (OR = 3.52E-19, 95% CI: 5.48E-32–2.26E-06, *p* = 0.005). Simultaneously, a relationship following the same trend was discerned through the weighted median method (OR = 6.59E-19, 95% CI = 3.36E-35–0.013, *p* = 0.029). These results were graphically represented in both the forest plot (Figure 2) and the scatter plot (Figure 3). Given that IVW and weighted median method hold an edge in preserving superior estimation precision over the MR-Egger method in MR analysis, the outcomes from the MR analysis provide support for a potential causal association between rosuvastatin use on bladder cancer.

Additionally, the MR-Egger regression intercept analysis (*p* = 0.901) and the MR-PRESSO global test (*p* = 0.487) did not show significant pleiotropy (Table 3). The Cochran Q-test results from both the MR-Egger (*p* = 0.285) and IVW (*p* = 0.410) methods also demonstrated no heterogeneity in our findings. The funnel plots were shown in Supplementary Figure S1. Furthermore, the leave-one-out analysis demonstrated that the causal association estimate was not influenced by the exclusion of any individual SNP (Supplementary Figure S2). These outcomes provide confidence in the validity and robustness of the causal inference derived from the MR analysis.

## 4 Discussion

In this research, we utilized a two-sample MR analysis based on GWAS summary-level data to assess the causal impact of statin therapy (atorvastatin, simvastatin, and rosuvastatin) on bladder cancer. The MR analysis indicated that rosuvastatin usage had a genetically determined causal effect on reducing bladder cancer risk. However, there were no causal effects on bladder cancer risk from the use of atorvastatin or simvastatin.

In the present epidemiological research, the influence of statins on cancer risk remains uncertain. A population-based, nested, case-control study with 3,129 patients and 16,976 controls from the PHARMO database was conducted to investigate the potential protective effect of statin therapy on cancer risk. This study reported a 20% reduction in cancer risk associated with statin use (adjusted odds ratio [OR], 0.80; 95% CI, 0.66–0.96) (Graaf et al., 2004). Additionally, a multi-regional observational study from the BioBank Japan cohort indicated that statin monotherapy was effective in reducing all-cause and cancer mortality (Yokomichi et al., 2017). In contrast, a matched case-control study using the General Practice Research Database observed no significant relationship between current statin use and the risk of 13 types of cancer (Kaye and Jick, 2004). Another investigation involving 4,913 cancer patients and 3,900 controls found no evidence to support a positive or negative association between statin use and 10 cancer types (Coogan et al., 2007).

Further research by Jun-Jun Yeh et al., utilizing Taiwan’s National Health Insurance Research Database, assessed the effects of statins on cancer risk among patients with interstitial lung disease (ILD) and pulmonary fibrosis. Their findings suggested a lower risk of bladder cancer associated with statin use in this population (Yeh et al., 2021). A multicenter study on patients with T1 high-grade non-muscle invasive urothelial bladder cancer showed that statin use was independently associated with a lower risk of recurrence (HR: 0.80, 95% CI: 0.67–0.95; *p* = 0.009), implying a beneficial effect on recurrence rates (Ferro et al., 2021). Additionally, a retrospective cohort study of individuals aged 66 and over, diagnosed with non-muscle invasive bladder cancer (NMIBC) between 1992 and 2012, demonstrated that statin users had better overall survival compared to nonusers (Richard et al., 2017). However, a population-based case-control study in Taiwan, including 325 bladder cancer cases and 1,300 controls, did not provide evidence supporting any beneficial or detrimental associations between statin use and bladder cancer risk (Kuo et al., 2012). A retrospective analysis of 1,117 patients treated with transurethral resection of the bladder (TURB) for NMIBC at three institutions from 1996 to 2007 assessed the impact of statin use on patient outcomes and the efficacy of intravesical BCG therapy. The findings indicated that statin use did not affect outcomes differently compared to non-use, nor did it impact the efficacy of BCG immunotherapy (Crivelli et al., 2013). And a meta-analysis including 10 studies found a non-significant increase in bladder cancer risk among statin users compared with non-users, and no association between statin use and BCa local control, recurrence, survival or mortality (Symvoulidis et al., 2023). Another meta-analysis including 13 studies also suggested that there was no association between statin use and risk of BCa(Zhang et al., 2013). It is noteworthy that these studies did not differentiate the effects based on specific statins.

Atorvastatin demonstrated notable antiproliferative and pro-apoptotic effects in human bladder cancer cells (Kamat and Nelkin, 2005). It also induced autophagy in these cells *in vitro*. Additionally, when used with autophagy inhibitors, atorvastatin’s cytotoxicity was enhanced, further promoting apoptotic cell death (Kang et al., 2014). In animal studies, Belmiro Parada et al. explored the chemopreventive efficacy of atorvastatin against nitrosamine-induced rat bladder cancer and observed a significant inhibitory impact on cancer development, likely due to its antioxidant, anti-proliferative, and anti-inflammatory actions (Parada et al., 2012). Despite these findings, no clinical trials have confirmed atorvastatin’s protective effect in bladder cancer patients. Similarly, our findings did not support a causal link between atorvastatin use and reduced bladder cancer risk.

Simvastatin inhibited bladder cancer cell metastasis by blocking epithelial-mesenchymal transition (EMT) and disrupting AKT/GSK3β pathways, while also suppressing cell proliferation and causing G1/G0 phase cell cycle arrest through the Peroxisome Proliferator-Activated Receptor (PPAR)γ signaling pathway (Wang et al., 2016). Additionally, the combination of simvastatin with romidepsin synergistically killed bladder cancer cells, with mechanisms involving ER stress induction, AMPK activation, histone acetylation, and enhanced PPARγ expression (Okubo et al., 2021). Moreover, Pleomorphic adenoma gene like-2 (PLAGL2) facilitated bladder cancer progression via RACGAP1/RhoA GTPase/YAP1 signaling, and its proproliferative and prometastatic effects were negated by the RhoA inhibitor simvastatin (Chen et al., 2023). Contrarily, a 10-year multicentric retrospective study in Lebanon established a duration-response relationship between simvastatin use and bladder cancer risk (OR = 1.189), revealing a detrimental link with the increased duration of simvastatin intake (Chalhoub et al., 2023). Our findings showed that there was no causal link between simvastatin use and bladder cancer risk among the European population.

Rosuvastatin triggered autophagic responses in human papillary thyroid cancer B-CPAP cells at lower doses, with a transition to apoptosis observed as rosuvastatin concentrations increased (Zeybek et al., 2011). When used alone or in a combined strategy with difluoromethylornithine, rosuvastatin significantly inhibited colon adenocarcinomas in male F344 rats induced by azoxymethane (AOM) and enhanced the functionality of natural killer (NK) cells (Janakiram et al., 2016). Furthermore, rosuvastatin prevented spheroid formation and epithelial-mesenchymal transition (EMT) in the prostate cancer PC-3 cell line (Deezagi and Safari, 2020) and exhibited antiangiogenic and antitumor properties that curtailed prostate cancer growth (Wang et al., 2010). A population-based cohort study from the Database of Clalit Health Services indicated that extended use of rosuvastatin was linked to a lower risk of prostate cancer (HR 0.22, 95% CI 0.08–0.75) (Lustman et al., 2014). In this study, MR analysis suggested that rosuvastatin use has a genetically determined causal effect in reducing bladder cancer risk.

Our research has several noteworthy strengths. Initially, we assessed the impact of three specific statins on bladder cancer risk using a two-sample MR analysis, potentially offering more profound insights for subsequent research. Given statins’ known benefits in lowering cardiovascular risk through their antidyslipidemic properties, their potential protective effects against bladder cancer could translate into significant medical and socioeconomic advantages for patients with common risk factors. Furthermore, Mendelian Randomization, which utilizes extensive data from Genome-Wide Association Studies (GWAS) to simulate a randomized controlled trial, offers a cost-effective alternative to observational studies and minimizes the risk of reverse causation. Additionally, the selection of our instrumental variable SNP, which occurs randomly at conception, helps eliminate confounding bias. Lastly, by choosing participants from the European demographic, we aimed to decrease potential biases stemming from population stratification.

This study is subject to several limitations that must be considered when interpreting and generalizing the results. Initially, while the study population addressed racial discrepancies, it remains uncertain if these findings can be extended to different racial groups and geographic areas. Additional GWAS studies across diverse regions might yield stronger evidence concerning the relationship between statin use and bladder cancer risk. Additionally, our reliance on summary-level data precluded the possibility of analyzing non-linear relationships or effects that vary across subgroups. Moreover, we accessed only the GWAS summary-level data for atorvastatin, simvastatin, and rosuvastatin, which limits our understanding of how other statins might affect bladder cancer risk. Then, the funnel plot for the instrumental variables is asymmetric, indicating bias and confounding in the findings. Lastly, the MR method used was restricted to establishing causal connections and did not allow for the investigation of the mechanisms underlying these associations, which would require more comprehensive studies.

## 5 Conclusion

In this study, we presented evidence supporting a potential genetically determined causal link between rosuvastatin use and a decreased risk of bladder cancer in the European population, using two-sample Mendelian Randomization analysis. The results indicated that rosuvastatin usage was linked to a lower risk of bladder cancer, while no significant genetically predicted causal effects were observed for atorvastatin or simvastatin. The results of this study were not sufficient to support the conclusion that statins were associated with low incidence of bladder cancer. Additional research into the mechanisms is required to elucidate the intricate relationship between statin treatment and bladder cancer risk.

## Data Availability

The original contributions presented in the study are included in the article/Supplementary Material, further inquiries can be directed to the corresponding authors.
