# Supplementary material for: Assessing the role of statin therapy in bladder cancer: evidence from a Mendelian Randomization study
Source: Front Pharmacol. 2024 Jul 19;15:1427318. doi: 10.3389/fphar.2024.1427318 (PMC11294080; doi:10.3389/fphar.2024.1427318)
Supplement: Supplementary file 1 [file Table2.DOCX]

Supplementary Material

# Supplementary Figures


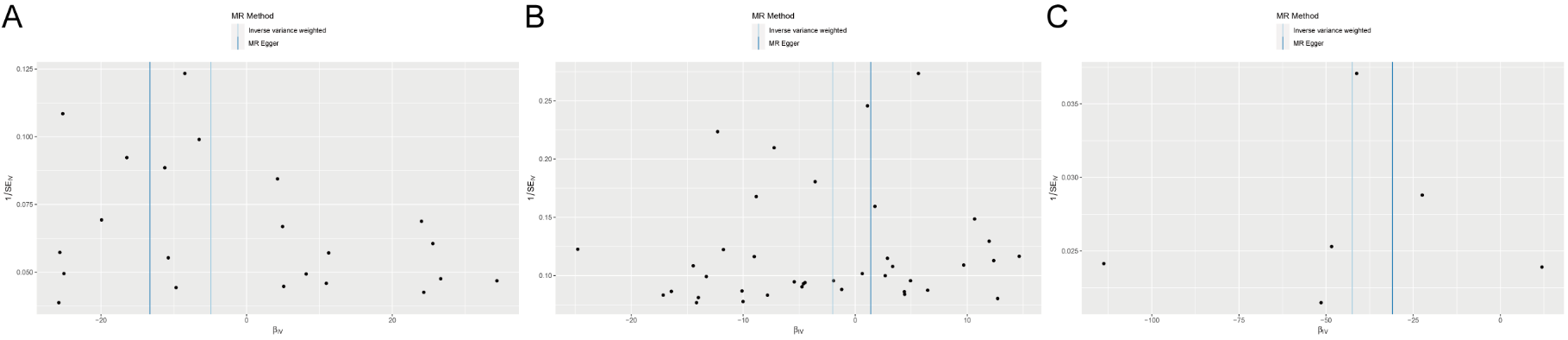


**Supplementary Figure 1** Funnel plot for instrumental variables of atorvastatin(A), simvastatin(B), and rosuvastatin (C) to assess heterogeneity.


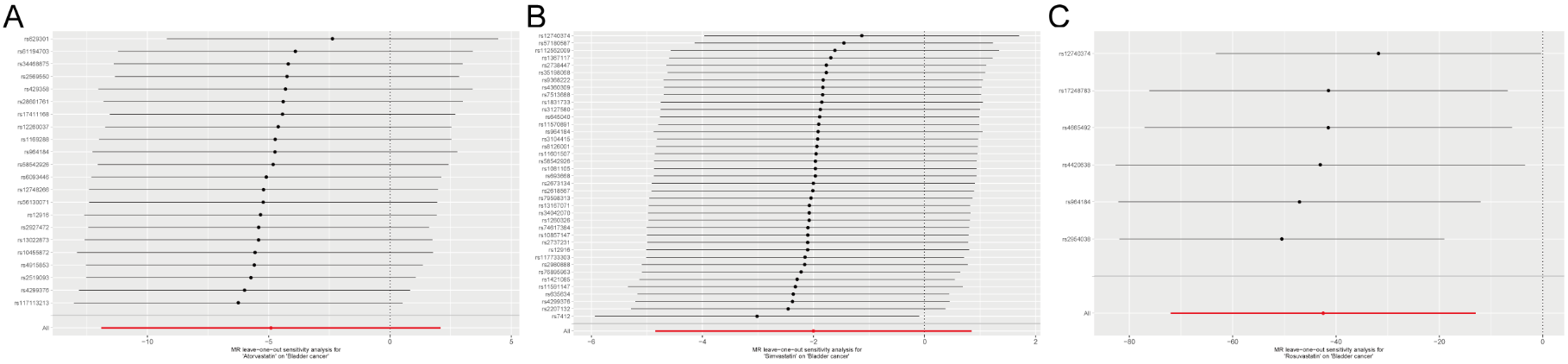


**Supplementary Figure 2** Leave-one-out analysis for the impact of individual SNPs on the association between atorvastatin(A), simvastatin(B), and rosuvastatin (C) use on bladder cancer. By leaving out exactly one SNP, it shows how each individual SNP influences the overall estimate
